# A Meta-Analysis on Vitamin D Supplementation and Asthma Treatment

**DOI:** 10.3389/fnut.2022.860628

**Published:** 2022-07-06

**Authors:** Meiqi Liu, Jun Wang, Xinrong Sun

**Affiliations:** ^1^Department of Respiratory Medicine, Xi’an Children’s Hospital, Xi’an Jiaotong University, Xi’an, China; ^2^Department and Institute of Infectious Disease, Xi’an Children’s Hospital, Xi’an Jiaotong University, Xi’an, China

**Keywords:** vitamin D, asthma, FEV1, asthma exacerbations, children

## Abstract

**Background:**

Vitamin D, as an immunomodulator, may be related to the therapeutic effect of asthma patients, but the research in this area is still controversial. The aim of this meta-analysis was to analyze the role of vitamin D supplementation in the treatment of asthma patients.

**Materials and Methods:**

Randomized Controlled Trials (RCTs) of vitamin D supplementation in asthma were searched in PubMed, EMBASE, and the Cochrane library. Primary outcomes were forced expiratory volume in one second (FEV1), asthma exacerbations, Asthma Control Test scores (ACT scores), and fractional exhaled nitric oxide (FENO).

**Results:**

A total of 10 RCTs were included, including 1,349 patients. Vitamin D supplementation didn’t affect the ACT scores (SMD = 0.04, 95% CI = −0.13 to 0.21, *P* = 0.87), FEV1 (SMD = 0.04, 95% CI = −0.35 to 0.43, *P* < 0.01) and FENO (SMD = −0.01, 95% CI = −0.22 to 0.20, *P* = 0.27), but reduced the rate of asthma exacerbations (RR = 0.69, 95% CI = 0.41 to 0.88, *P* < 0.01), especially in subgroups of children (RR = 0.46, 95% CI = 0.30 to 0.70, *P* = 0.83) and follow up time less than 6 months (RR = 0.45, 95% CI = 0.32 to 0.63, *P* = 0.95). Additionally, though there was only one study included in the subgroup, it significantly enhanced FEV1 at the last visit for patients whose FEV1 baseline value was less than 70% (SMD = 0.94, 95% CI = 0.47 to 1.41).

**Conclusion:**

Vitamin D supplementation can reduce asthma exacerbations, especially in children, and within 6 months of follow up time. In addition, vitamin D has a positive effect on improving FEV1 of patients whose FEV1 baseline value is less than 70%, but more RCTs are still needed to support this conclusion.

**Systematic Review Registration:**

[https://inplasy.com], identifier [10.37766/inplasy20 22.6.0049].

## Introduction

As one of the most common chronic, non-communicable diseases, asthma is a heterogeneous clinical syndrome affecting approximately 334 million people worldwide (1). It is defined by Expert Panel Report 3 (EPR-3) as “a chronic inflammatory disorder of the airways in which many cells and cellular elements play a role: in particular, mast cells, eosinophils, neutrophils (especially in sudden onset, fatal exacerbations, occupational asthma, and patients who smoke), T lymphocytes, macrophages, and epithelial cells. In susceptible individuals, this inflammation causes recurrent episodes of coughing (particularly at night or early in the morning), wheezing, breathlessness, and chest tightness. These episodes are usually associated with widespread but variable airflow obstruction that is often reversible either spontaneously or with treatment” (2). The global prevalence of asthma in adults is 4.3% (3) but varies in different countries, 7.8–11.9% in the United States (4–6), 10% in Japan (7), 2.38% in India (8), and 1.2–5.8% in China (9). More than 400 thousand people were estimated by the Global Burden of Disease collaboration to die from asthma, mainly in low- and middle-income countries (10). Airflow limitation, an important feature of asthma, is more common in low-and middle-income countries due to the higher prevalence of known risk factors and poor asthma management compared to high-income countries (11). Asthma in children is predominantly male, whereas in adults it is the opposite, probably due to the effects of sex hormones (12).

The existent evidence indicates that asthma is a disease associated with various factors, including environmental factors [air pollution (13), climate change, pollen (14), microbial exposure (15), and allergic triggers (16)], host factors [nutrition state (17) and infection (18)], and genetic factors [genetic susceptibility sites of asthma (19)]. Notably, many studies have shown that dietary factors could affect the course and development of asthma. High consumption of vegetables and fruits (20–23), especially apples and oranges, could reduce the risk of asthma. Pro-inflammatory cytokines associated with fruit and vegetable intake were simultaneously decreased and anti-inflammatory factors were increased (24, 25). In addition, there was a positive association between the frequent consumption of dairy products with asthma (26) and bronchial hyperreactivity (27). However, acute effects of milk ingestion were not significant in asthma patients (28–30). A diet that emphasizes fruits, vegetables, and whole grains, but not high-fat meat and dairy products, was related to reducing the risk of asthma (31–33).

As one of the fat-soluble vitamins required by the human body, vitamin D is obtained mainly through the skin synthesis pathway after ultraviolet B (UVB) radiation, and a small part from food (oily fish, egg yolk, mushroom, liver, or organ meat) and supplements. Cholecalciferol (vitamin D3) is derived from animals and ergocalciferol (vitamin D2) is derived from plants (34). Recently, vitamin D deficiency, one of the major risk factors in asthma, has triggered more and more interest in research, which was confirmed to involve in the development and prognosis of a variety of diseases, including cancer (35), inflammatory bowel disease (36), urinary tract infection (37), respiratory infections (38), and asthma (39). It was reported that the risk of acute respiratory infection (ARI) was reduced in individuals with high serum 25(OH)D levels (40). What’s more, a case-control study has reported that children who require hospitalization for acute respiratory infections had a significantly higher risk of vitamin D deficiency than children with mild acute respiratory infections (41). 1,25 (OH) 2D exerts antiviral activity and regulates inflammatory response to viral infection by stimulating cathelicidin release, regulating toll-like receptor expression, and inhibiting pro-inflammatory cytokines production (42). An RCT has proved that supplementation of vitamin D could protect against the development of acute respiratory tract infection (43). As for the rise of the COVID-19 pandemic, calcitriol non-significantly suppressed the expression of angiotensin II (Ang II) receptor type 1 (AT1) and angiotensin-converting enzyme (ACE), but markedly reduced Ang II formation, which acts as host cell receptors mediating SARS-CoV-2 infection (44). Evidence showed that vitamin D supplementation might reduce the risk of infection and death in COVID-19 (45, 46).

Furthermore, respiratory tract infection is the main cause of asthma aggravation (47). A great many studies have found that patients with low vitamin D levels were more likely to have asthma exacerbations (48–50). In addition, there is sufficient evidence that exposure to tobacco smoke and nicotine during the prenatal and postnatal periods impairs lung development, alters the immune response to viral infection, and increases the prevalence and severity of childhood wheezing (51). Chinellato I’s research demonstrated that vitamin D levels were significantly higher in children with non-smoking parents than those with both smoking parents, and were intermediate in those exposed to single maternal or paternal smoking (52). It has been reported that a modest reduction in 25- hydroxyvitamin D in pregnant women exposed to cigarette smoke, is probably because of the reduced ability of the placenta of women who smoke to transport vitamin D (53). In addition, smoking in adults was associated with osteopenia and decreased serum 25(OH)D and parathyroid hormone (PTH) concentrations (54, 55). While for smokers, Ben Michael Brumpton’s team found that Low serum 25(OH)D levels had a weaker correlation with greater decreases in lung function in adults with asthma, and a stronger correlation was observed in non-smokers, but not in ever smokers (56). As for the effect of vitamin D supplementation in smokers or non-smokers with asthma, Sluyter J. D.’s study demonstrated that vitamin D supplementation significantly improved the lung function of both ever-smokers and non-smokers with asthma. However, there is still a lack of RCTs on vitamin D supplementation in patients with asthma varying by smoking status (57).

However, there are contradictions between the mechanism research and clinical prognosis research on the effect of vitamin D supplementation on asthma. Some research has determined the relationship between vitamin D deficiency and the overall worsening of lung function and symptoms in patients with asthma (39, 58, 59). In terms of mechanism research, some asthma mouse model studies have indicated the protective effect of vitamin D supplementation. Serum IgE, whose elevated expression is the characteristic of active airway inflammation (60), could be reduced significantly via vitamin D supplementation. What’s more, vitamin D exerted a protective effect by reducing airway remodeling and inhibited airway inflammation by reducing oxidative stress and regulating the Th17/Treg balance and the NF-κB pathway (61). The classical Wnt/β-catenin pathway plays a key role in cell proliferation, cell migration, stem cell self-renewal, organogenesis, tissue homeostasis under physiological conditions, and damaged tissue repair (62). The intracellular accumulation and nuclear transfer of Wnt/β-catenin have a great impact on the maturation and structural adaptation of the lung, including the development of airway smooth muscle precursor cells, the maintenance of airway smooth muscle growth, and the regulation of its contraction, which was related to the pathogenesis of asthmatic airway remodeling (63–65). And the research showed that vitamin D improved airway remodeling in asthma by down-regulating the activity of the Wnt/β-catenin signaling pathway (66). In contrast, vitamin D deficiency aggravated the progression of asthma by increasing eosinophils, decreasing T regulatory cells, increasing NF-κB expression, and increasing pro-inflammatory cytokines (67). So far, there have been a number of meta-analyses regarding vitamin D supplementation in relation to asthma treatment. Some meta-analysis (68–72) have manifested that vitamin D supplementation reduced the rate of asthma exacerbations for patients with systemic corticosteroid treatment, especially in patients with vitamin D insufficiency, but didn’t affect the lung function (FEV1 or FENO) and ACT scores. However, there are still a few clinical studies manifesting that vitamin D supplementation in vitamin D-deficient patients didn’t improve the course and development of asthma (73, 74). Asthma control, asthma exacerbations, and lung function were all unaffected by vitamin D supplementation. The conclusions are not uniform, and some study populations only include children or adults. Therefore, a systematic meta-analysis of Randomized Controlled Trials (RCTs) was conducted to investigate the role of vitamin D supplementation and asthma treatment.

## Objectives

The aim of this study was to evaluate the effect of vitamin D supplementation on clinical outcomes (Asthma Control Test scores, ACT scores; forced expiratory volume in 1 s, FEV1; fractional exhaled nitric oxide, FENO; asthma exacerbations) in asthma patients.

## Methodology

Preferred reporting items (PRISMA) statements of systematic review and meta-analysis were used for the meta-analysis (75).

### Search Strategy

A comprehensive literature search using predefined keywords from articles published over the last decade was conducted on PubMed, EMBASE, and the Cochrane library.

Manually search to retrieve articles using keywords: {(Asthma [Title/Abstract]) OR (asthma exacerbations [Title/Abstract])} AND (vitamin D [Title/Abstract]) AND (supplementation [Title/Abstract]) AND (RCTs [Title/Abstract]).

### Inclusion Criteria

Randomized Controlled Trials published in English were included, in which vitamin D was prospectively added after the diagnosis of asthma to explore the role of vitamin D supplementation in asthmatics. The intervention group consisted of asthma patients who received any form or dose of vitamin D supplementation in addition to standard treatment, while those who did not receive vitamin D formed the control group. Then, the asthma-related outcomes were analyzed, including lung function (FEV1), FENO, ACT scores, and the rate of asthma exacerbations.

### Exclusion Criteria

Retrospective and observational studies, articles or preprints not published in peer-reviewed journals, articles that did not mention the results included in our study or for which the data were incomplete, and retrospective vitamin D supplementation studies were excluded.

### Study Selection

All studies selected from the database were filtered by title and abstract to exclude unrelated or duplicate articles. Two authors screened independently, and a third co-author was involved in resolving differences that arose during the literature screening process.

### Data Extraction

Two authors independently extracted the relevant data from the article, including study population (age, country), intervention measures (vitamin D administration method and dose), follow-up time and outcomes (FEV1, FENO, asthma exacerbations and ACT scores), and baseline data related to the results (mean age, FEV1, ACT scores and vitamin D content).

### Quality Assessment

The two authors independently evaluated the methodological quality of the included studies based on Cochrane’s systematic review guidelines and resolved the differences through discussion with the third co-author. The risk of bias was plotted using Review Manager 5.4 and individual quality analysis was performed using the GRADE-PRO method.

### Statistical Analysis

In this meta-analysis, we used risk ratio (RR) and standard error (SMD) as the impact measurement standards, R software version 4.1.1 (R project in Vienna, Austria) for statistical analysis and forest mapping. The methodological quality of the study was evaluated using Review Manager Version 5.4 following the Cochrane guidelines. A random effect model was used for statistical analysis due to differences in the mix of interventions and participants. The heterogeneity among studies was assessed by Cochran Q-test, and *P* < 0.05 was considered statistically significant. When data from three or more studies were available, results were summarized using either the standardized mean difference (SMD) for continuous variables or the risk ratio (RR) for dichotomized variables. Statistical analysis was performed using the Mann-Whitney *U* test, and a two-sided *P*-value of <0.05 was considered statistically significant. Using the I^2^ statistic to evaluate the degree of heterogeneity between included studies. I^2^ values of 25, 50, and 75% were considered low, medium, and high heterogeneity. In addition, in order to explore the impact and heterogeneity of each outcome, prespecified subgroup analyses were stratified by FEV1 baseline values (less than 70% or greater), age (children or adults), and follow up time. The use of funnel plots failed to demonstrate potential publication bias since each result did not reach 10 studies. Sensitivity analyses were performed to check the robustness of the results by omitting one study and analyzing the remainder in each round.

## Results

### Study Characteristics

**FIGURE 1:**
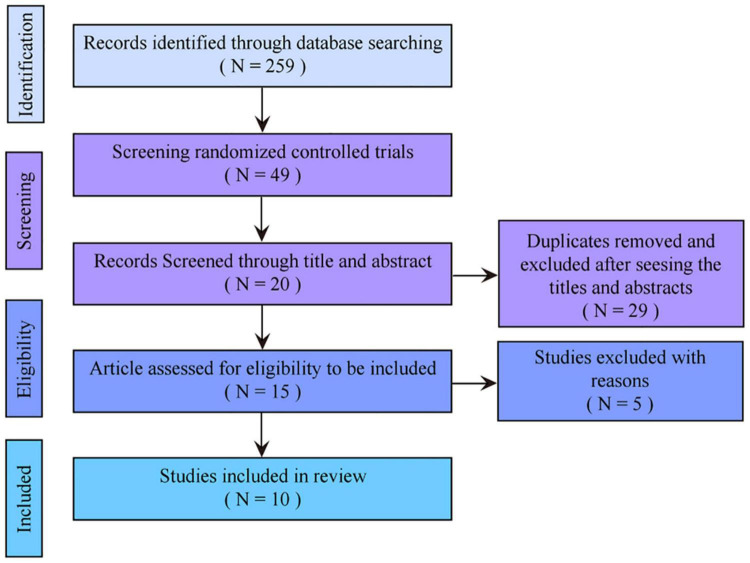
Study flow diagram.

In this review, we used database search and a comprehensive manual search strategy. A total of 259 studies was found in the initial search, and 49 RCTs were screened out. After manual deletion of duplicate references, the remaining 20 studies were selected by title and abstract. There were 15 eligible articles after excluding irrelevant articles. Among them, studies in which outcome indicators were variation quantity before and after intervention or the outcome indicators which had missing values were excluded. Eventually, 10 studies were included in the review and met the inclusion criteria through evaluating the full text (Figure 1 and Table 1).

**TABLE 1:** Summary of the included articles in this review.

References	Study design	Country	Age	Sample size (I/C)	Participants	Basic treatment	Intervention	Control/ Placebo	Follow-up time	Outcomes
Majak et al. (80)	Randomized, double-blind, parallel-group trial	Poland	5–18 years old	24/24	Patients with newly diagnosed asthma and sensitive only to house dust mites	Budesonide 800 mg/d	Vitamin D-500 IU Cholecalciferol	Placebo.	2 months 4 months	FEV1, the rate of patients with asthma exacerbations.
Castro et al. (76)	Randomized, double-blind, parallel-group study	United States	≥18 years old	201/207	Participants with asthma and a serum 25-hydroxyvitamin D level of less than 30 ng/mL	Inhaled ciclesonide (320 μg/day) and levalbuterol	Vitamin D3 100 000 IU once, then 4,000 IU/day for 28 weeks	Placebo.	3 months 5 months 7 months	The overall exacerbation rate.
Yadav et al. (83)	Randomized, double-blind, placebo-controlled trial	India	3–14 years old	50/50	Children with moderate to severe asthma as per GINA guidelines	Steroid (As one of the outcomes, the dose is not constant)	Vitamin D3 (Cholecalciferol) 60,000 IU per month	Placebo powder in the form of glucose sachet	1 month 2 months 3 months 4 months 5 months 6 months	Number of exacerbations
de Groot et al. (77)	Randomized, double-blind, placebo-controlled trial	Holland	≥18 years old	22/22	Patients with asthma	Budesonide (400–800 μg/day)	Single high dose of long-acting oral vitamin D3 preparation (400,000 IU)	Placebo	1 week 9 weeks	FEV and FENO
Martineau et al. (78)	Randomized, double-blind, placebo-controlled trial	United Kingdom	16–80 years old	125/125	Patients with asthma	Inhaled corticosteroids, long-acting β-2 agonist, oral corticosteroids (The details are unknown and the dose is not constant)	Six 2-monthly oral doses of 3 mg vitamin D3	Placebo	2 months 6 months 12 months	Severe asthma exacerbation, ACT score, FEV1, and FENO
Musharraf et al. (81)	Randomized controlled trial	Pakistan	16–46 years old	40/40	Patients were diagnosed of bronchial asthma for at least 1 year with vitamin D levels less than 30 ng/ml	Salmeterol/ fluticasone inhaler preparation Salmicort^§^ 25/250 μg twice daily, Montelukast Montika^§^ 10 mg at night	Vitamin D3 50,000 units fortnightly for a period of 3 months in addition to standard treatment	Standard treatment	3 months	Asthma exacerbations.
Dodamani et al. (74)	Randomized, parallel-group study	India	≥12 years old	15/15	Patients with ABPA complicating asthma	Oral prednisolone 0.5 mg/kg/day for 4 weeks. Prednisolone was then tapered by 5 mg every 2 weeks and discontinued.	Vitamin D3 60,000 IU once weekly for 8 weeks	Placebo	2 months 4 months 6 months	Number of asthma exacerbations
Shabana et al. (79)	Double blinded randomized controlled interventional study	Egypt	≥19 years old	42/37	Patients with asthma	Inhaled corticosteroids (fluticasone, budesonide, and ciclesonide), leukotriene antagonist (montelukast), long-acting beta agonists (salmeterol and formoterol), and theophylline (The details are unknown)	Single dose of 300,000 IU of vitamin D3.	Placebo	3 months	FEV1.
Jat et al. (73)	Randomized, double-blind, placebo-controlled trial	India	4–12 years old	125/125	Patients with asthma	Inhaled corticosteroids, long-acting β-2 agonist, oral corticosteroids (The details are unknown and the dose is not constant)	Vitamin D orally 1,000 IU/day for 9 months.	Placebo	9 months	CACT score, FEV1.
Thakur et al. (82)	Randomized, blinded, parallel-group, placebo-controlled trial	India	6–11 years old	30/30	Patients with moderate persistent asthma	Inhaled corticosteroids, long-acting β-2 agonist, systemic steroid, leukotriene receptor antagonist (The details are unknown)	Vitamin D orally 2,000 IU/day	Placebo	3 months	CACT score, FEV1, FeNO, and Number of patients with exacerbation

### Description of the Included Studies

The characteristics and baseline data of included RCTs were presented in Tables 1, 2 In this review, all the included studies were RCTs, including the detailed information of 1,349 subjects, with the sample size ranging from 15 to 207, and the locations of the subjects involved in the United States (76), Holland (77), United Kingdom (78), Egypt (79), Poland (80), Pakistan (81), and India (73, 74, 82, 83).

**TABLE 2:** Baseline characteristics of patients in the eight studies included.

References	Age (years) Mean (SD)		FEV1% Mean (SD)		FENO Mean (SD)		ACT score Mean (SD)		25-hydroxyvitamin D Mean (SD)	
	I	C	I	C	I	C	I	C	I	C
Majak et al. (80)	10.8 (3.2)	11.1 (3.3)	94.4 (13)	98.7 (12)	NA	NA	NA	NA	NA	NA
Castro et al. (76)	39.9 (13.1)	39.5 (12.7)	91.32 (13.83)	92.09 (13.65)	NA	NA	19.33 (3.73)	19.67 (3.73)	19.8 (7.8)	18.6 (7.7)
Yadav et al. (83)	9.15 (2.444)	10.00 (1.876)	NA	NA	NA	NA	NA	NA	NA	NA
de Groot et al. (77)	59.0 (9.7)	53.6 (16.7)	99.1 (15.7)	97.6 (18.1)	26.33 (9.51)	38.33 (41.21)	NA	NA	24.9 (9.9)	22.3 (9.5)
Martineau et al. (78)	49.4 (14.8)	46.4 (13.8)	82.0 (18.7)	81.0 (20.4)	38.1 (29.1)	37 (26)	19.2 (3.9)	18.9 (3.9)	19.97 (10.10)	19.81 (9.70)
Musharraf et al. (81)	29.70 (7.74)	29.43 (8.47)	NA	NA	NA	NA	NA	NA	NA	NA
Dodamani et al. (74)	33 (12.5)	32 (12.2)	NA	NA	NA	NA	NA	NA	23.07 (29.04)	20.97 (29.2)
Shabana et al. (79)	34.00 (7.40)	35.50 (7.00)	68.38 (12.00)	67.54 (9.93)	NA	NA	NA	NA	17.56 (2.74)	18.16 (2.89)
Jat et al. (73)	8.2 (2.3)	7.8 (2.2)	92.5 (21.7)	97.0 (17.5)	NA	NA	21.7 (4.2)	21.9 (3.6)	11.6 (4.6)	10.8 (4.4)
Thakur et al. (82)	9.0 (1.7)	8.7 (1.6)	75.3 (26.5)	75.6 (15.7)	19.77 (16.11)	22.27 (24.29)	18 (2.9)	15.5 (2.7)	15.8 (8.2)	16.5 (9.9)

Among the 10 included studies, one RCT (74) included patients with allergic bronchopulmonary aspergillosis (ABPA) complicating asthma, whereas the other nine RCTs included patients with asthma (73, 76–81) or moderate persistent asthma (82, 83). In addition, six studies in which participants were adults (74, 76–79, 81), while the other four RCTs were children (73, 80, 82, 83).

There was significant heterogeneity in the doses of vitamin D used in the intervention groups, with the control group receiving an equal dose of placebo, and both two groups receiving a standardized treatment, inhaled corticosteroid, according to the guidelines. The follow-up time ranged from 1 week (77) to 12 months (78).

Two RCTs (78, 82) analyzed ACT score, asthma exacerbations, FENO, and FEV1 as outcome measures. The other three studies all analyzed FEV1 as the outcome in addition to ACT scores (73), FENO (77), and asthma exacerbations (80), respectively. Asthma exacerbations were used as an outcome in Castro’s (76), Dodamani’s (74), Yadav’s (83), and Musharraf’s (81) studies. The rest of one RCT (79) used FEV1 to evaluate the outcome of the two groups.

Baseline FEV1 values were reported in seven studies in the two groups, six of which were greater than 70% (73, 76–78, 80, 82), whereas one of which was less than 70% (79). Three RCTs reported FENO baseline values, two of which were higher than those in the intervention group (77, 82), and the other was the opposite (78). Four RCTs counted the baseline values of ACT scores, among which the median value of three RCTs was greater than 19 points (73, 76, 78) and the other was less than 19 points (82). Baseline data for 25-hydroxyvitamin D were available for seven RCTs enrolled, with all the studies less than 30 ng/ml, and two of them more than 20 ng/ml (74, 77) and the others less than 20 ng/ml (73, 76, 78, 79, 82) (Table 2).

### Methodological Quality of Study

**FIGURE 2:**
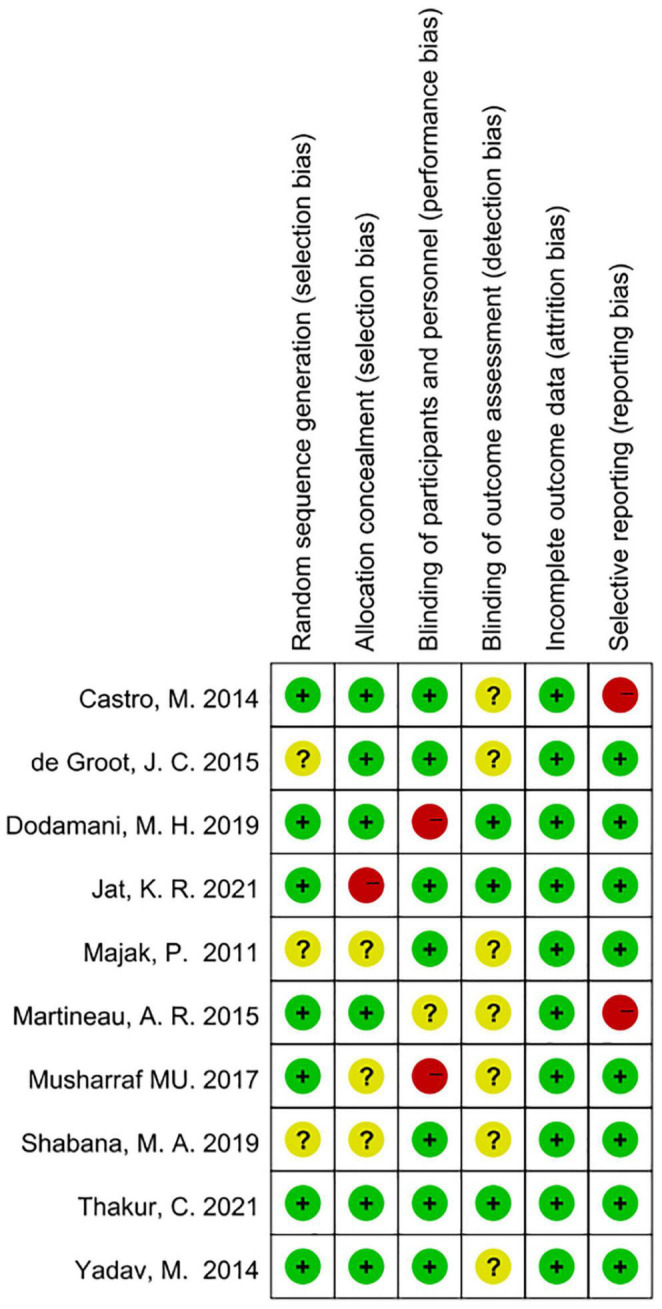
Risk of bias summary based on Cochrane Systematic Review Guidelines for each included study included in this review (green for low risk of bias, yellow for unclear risk of bias and red for high risk of bias).

**FIGURE 3:**
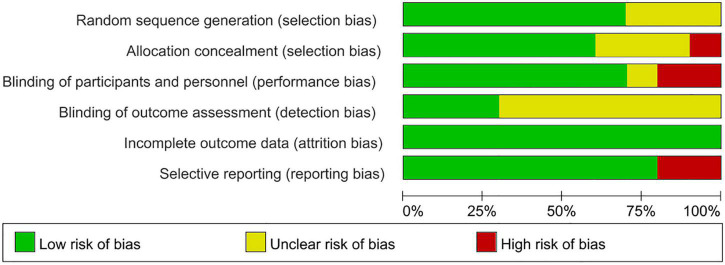
Risk of bias graph review authors judgments about each risk of bias item presented as percentages across various study designs.

According to Cochrane system evaluation guidelines, we conducted a risk bias assessment for each study included in this evaluation. A summary chart of bias risk was shown in Figure 2, in which red represents high deviation risk, green represents low deviation risk, and yellow represents ambiguous deviation risk. Figure 3 showed the risk of bias graph, in which the authors expressed our judgments on various risk items of bias in each study in percentage form.

Grade summary Table 3 gave an overall rating for the quality of evidence regarding the role of vitamin D supplementation in asthma patients. The GRADE summary demonstrated that the evidence for exacerbation of asthma (in the adult and over 6 months of follow-up subgroup) and FEV1 (in children, adults, and under 6 months of follow-up subgroup) were very low, meaning that the effect estimation was uncertain. It might be related to the significant difference in the dose and mode of vitamin D administration and the baseline data of patients across different RCTs.

**TABLE 3:** The overall rating for the quality of evidence profile for asthma related health outcomes based on the grading of Recommendations Assessment, Development, and Evaluation (GRADE) Working group methodology.

Certainty assessment							No. of patients		Effect		Certainty	importance
No. of studies	Study design	Risk of bias	Inconsistency	Indirectness	Imprecision	Other considerations	Vitamin D	Placebo	Relative (95% CI)	Absolute (95% CI)		
**Asthma exacerbations**												
7	Randomized trails	Serious^a^	Serious^b^	Serious^c^	Not serious	None	109/466 (23.4%)	163/478 (34.1%)	RR 0.59 (0.39 to 0.89)	140 fewer per 1,000 (from 208 fewer to 38 more)	Very low	Critical
**Asthma exacerbations children**												
3	Randomized trails	Not serious	Not serious	Serious^c^	Serious^d^	None	22/102 (21.6%)	48/102 (47.1%)	RR 0.46 (0.30–0.70)	254 fewer per 1,000 (from 329 fewer to 141 fewer)	Low	Critical
**Asthma exacerbations adults**												
4	Randomized trails	Serious^a^	Serious^b^	Serious^c^	Not serious	None	87/364 (23.9)	115/376 (30.6%)	RR 0.68 (0.40–1.18)	98 fewer per 1,000 (from 184 fewer to 55 more)	Very low	Critical
**Asthma exacerbations follow up time <6 months**												
5	Randomized trails	Serious^a^	Not serious	Serious^c^	Not serious	None	31/157 (19.7%)	71/157 (45.2%)	RR 0.45 (0.32–0.63)	249 fewer per 1,000 (from 308 fewer to 167 fewer)	Low	Critical
**Asthma exacerbations follow up time >6 months**												
2	Randomized trails	Serious^a^	Serious^b^	Serious^c^	Not serious	None	78/309 (25.2%)	92/321 (28.7%)	RR 0.87 (0.49–1.52)	37 fewer per 1,000 (from 146 fewer to 149 more)	Very low	Critical
**ACT score**												
3	Randomized trails	Serious^a^	Not serious	Serious^c^	Not serious	None	261	261	–	SMD 0.04 higher (0.13 lower to 0.21 higher)	Low	Critical
**FENO**												
3	Randomized trails	Serious^a^	Not serious	Serious^c^	Not serious	None	175	175	–	SMD 0.63 higher (4.77 lower to 6.03 higher)	Low	Critical
**FEV1**												
6	Randomized trails	Serious^a^	Serious^b^	Serious^c^	Not serious	None	331	320	–	SMD 0.04 higher (0.13 lower to 0.21 higher)	Low	Critical
**FEV1 children**												
3	Randomized trails	Serious^a^	Not serious	Serious^c^	Serious^d^	None	142	136	–	SMD 0.29 lower (0.52 lower to 0.05 lower)	Very low	Critical
**FEV1 adults**												
3	Randomized trails	Serious^a^	Serious^b^	Serious^c^	Not serious	None	189	184	–	SMD 0.39 higher (0.17 lower to 0.95 higher)	Very low	Critical
**FEV1 follow up time <6 months**												
4	Randomized trails	Not serious	Serious^b^	Serious^c^	Serious^d^	None	116	111	–	SMD 0.13 higher (0.51 lower to 0.77 higher)	Very low	Critical
**FEV1 follow up time >6 months**												
2	Randomized trails	Serious^a^	Not serious	Serious^c^	Not serious	None	215	209	–	SMD 0.12 higher (0.07 lower to 0.31 higher)	Low	Critical
**FEV1 baseline <70%**												
1	Randomized trails	Not serious	Not serious	Serious^c^	Not serious	None	42	37	–	SMD 0.94 higher (0.47 higher to 1.41 higher)	Moderate	Critical
**FEV1 baseline =V0%**												
5	Randomized trails	Serious^a^	Not serious	Serious^c^	Not serious	None	289	283	–	SMD 0.12 lower (0.33 lower to 0.10 higher)	Low	Critical

*^a^Some concern with method of randomization used, allocation concealment, binding of participants, binding of outcome assessment or selective reporting.*

*^b^Inconsistency was reported by moderate to high heterogeneity.*

*^c^There were differences in the follow up time points to measure the outcomes and vitamin D dosages and duration.*

*^d^The total sample size was less than 300.*

### Efficacy Outcomes

#### Asthma Control Test Scores

**FIGURE 4:**
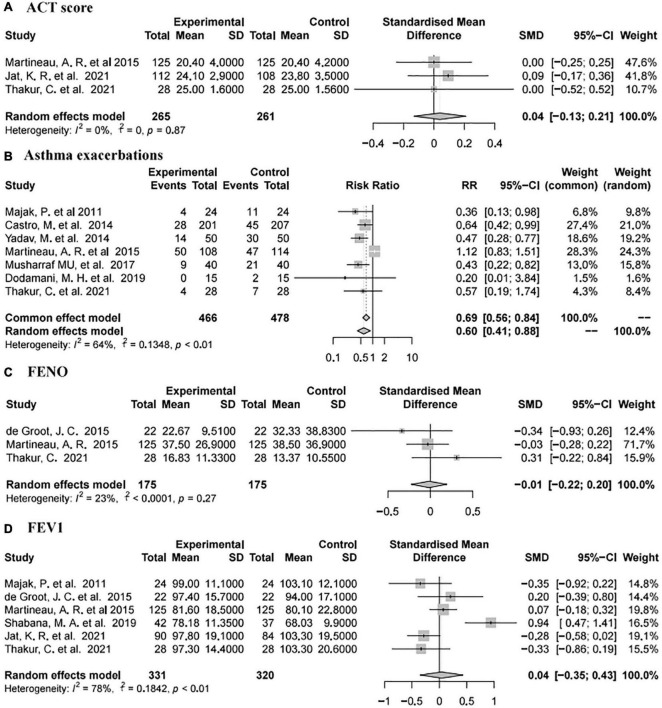
Forest plot random effect model for vitamin D supplementation for various outcomes.

Asthma Control Test (ACT) scores were reported in three studies (73, 78, 82) involving 526 individuals (265 intervention and 261 placebo). The pooled data demonstrated that there was no significant difference between the placebo and vitamin D groups (SMD 0.04, 95% CI −0.13 to 0.21, low heterogeneity (I^2^ = 0%, *P* = 0.87; Figure 4A).

#### Forced Expiratory Volume in One Second

Forced expiratory volume in one second was reported in six studies (73, 77–80, 82) involving 651 subjects (331 intervention and 320 placebo). The summary data showed that there was no significant difference between the placebo group and vitamin D group [SMD 0.04, 95% CI −0.35 to 0.43, high heterogeneity (I^2^ = 78%, *P* < 0.01; Figure 4D)].

**FIGURE 5:**
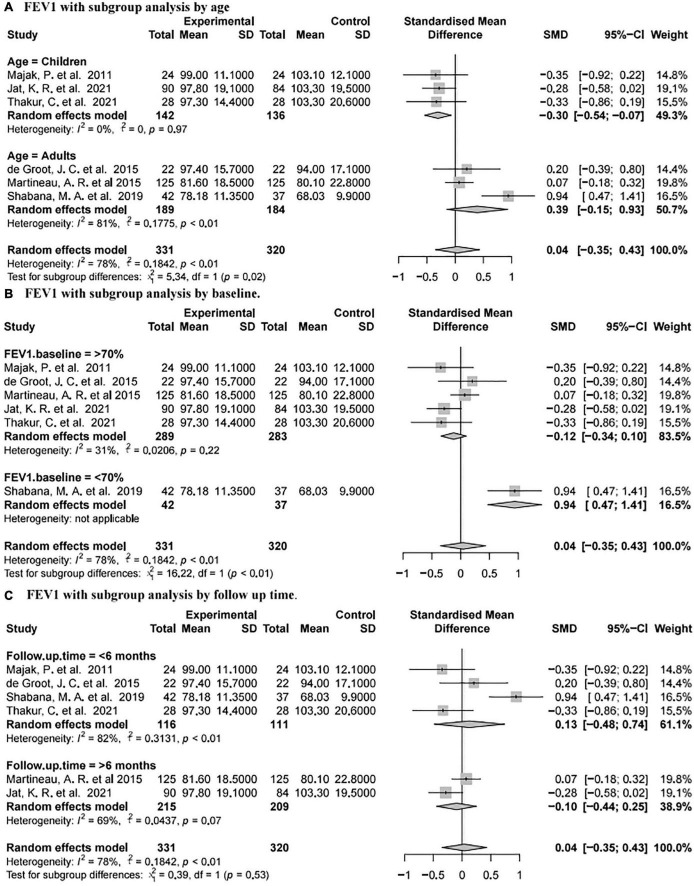
Forest plot random effect model for vitamin D supplementation for FEV1 with subgroup by various factors.

Subgroup analysis of the results for FEV1 was further performed (Figure 5). For the age subgroups, there was no significant difference between the placebo and vitamin D groups in adults [SMD 0.39, 95% CI −0.15 to 0.93, high heterogeneity (I^2^ = 81%, *P* < 0.01)], while vitamin D supplementation was associated with a reduction of FEV1 at the last visit in children [SMD −0.3, 95% CI −0.54 to −0.07, low heterogeneity (I^2^ = 0%, *P* = 0.97; Figure 5A)]. Regarding different FEV1 baseline values, there was no significant difference between the two groups for patients with FEV1 baseline values exceeding 70% [SMD −0.12, 95% CI −0.34 to 0.10, low heterogeneity (I^2^ = 31%, *P* = 0.22)], while vitamin D supplementation was related to the increase of FEV1 at last visit for patients with FEV1 baseline values less than 70% (SMD 0.94, 95% CI 0.47 to 1.41, without applicable heterogeneity; Figure 5B). For different follow-up times, vitamin D supplementation was not associated with FEV1 when the follow-up time was less than 6 months [SMD 0.13, 95% CI −0.48 to 0.74, high heterogeneity (I^2^ = 82%, *P* < 0.01)] or more than 6 months [SMD 0.11 95% CI −0.35 to 0.43, low heterogeneity (I^2^ = 39%, *P* = 0.53; Figure 5C)].

#### Asthma Exacerbations

Seven studies (74, 76, 78, 80–83) reported asthma exacerbations involving 944 subjects (466 intervention and 478 placebo). The pooled data showed that vitamin D supplementation was associated with a reduced rate of asthma exacerbations (RR 0.60, 95% CI 0.41–0.88, high heterogeneity (I^2^ = 64%, *P* < 0.01; Figure 4B)].

**FIGURE 6:**
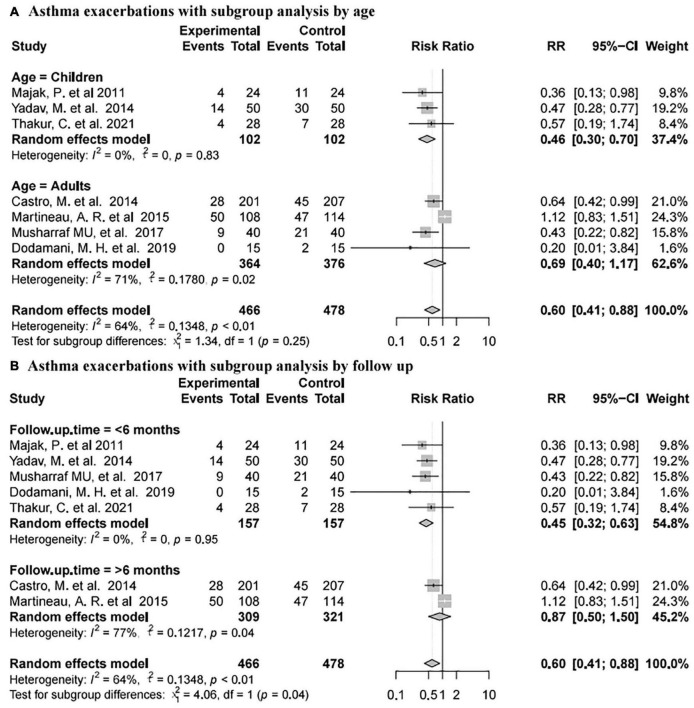
Forest plot random effect model for vitamin D supplementation for asthma exacerbations with subgroup by various factors.

Subgroup analysis of asthma exacerbation results was complicated (Figure 6). In terms of different age groups, there was no significant difference between the placebo and vitamin D groups in adults [RR 0.69, 95% CI 0.40 to 1.17, high heterogeneity (I^2^ = 71%, *P* = 0.02)], while vitamin D supplementation was related to reducing the rate of asthma exacerbations in children [RR 0.46, 95% CI 0.30 to 0.70, low heterogeneity (I^2^ = 0%, *P* = 0.83; Figure 6A)]. According to different follow-up time, vitamin D supplementation was related to the reduction of asthma exacerbations with less than 6 months of follow-up [RR 0.45, 95% CI 0.32 to 0.63, low heterogeneity (I^2^ = 0%, *P* = 0.95)], but not with more than 6 months of follow-up [RR 0.87, 95% CI 0.50 to 1.50, high heterogeneity (I^2^ = 77%, *P* = 0.04; Figure 6B)].

#### Fractional Exhaled Nitric Oxide

Fractional exhaled nitric oxide was reported in three studies (77, 78, 82) involving 350 subjects (175 intervention and 175 placebo). The pooled data indicated that there was no significant difference between the placebo and vitamin D groups [SMD −0.01, 95% CI −0.22 to 0.2, low heterogeneity (I^2^ = 23%, *P* = 0.27; Figure 4C)].

#### Sensitivity Analysis

**FIGURE 7:**
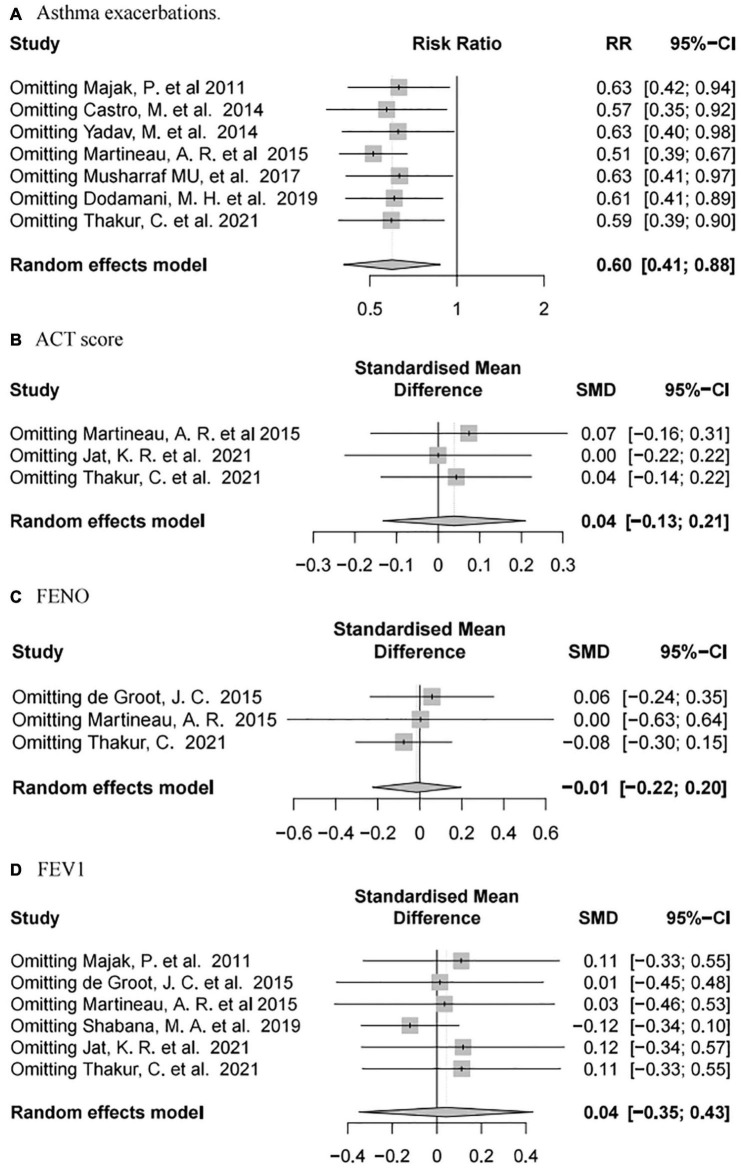
Forest plot random effect model of sensitivity analysis for vitamin D supplementation for various outcomes.

Sensitivity analysis of the outcomes using R language software (4.1.1) indicated that, after omitting each individual study, our results were consistent with the complete analysis of all endpoints, and that there was no significant correlation between vitamin D supplementation and the prognosis of patients with asthma (Figure 7).

## Discussion

In this systematic meta-analysis, vitamin D supplementation in asthmatics did not improve major health outcomes including ACT scores, FEV1, FENO, and asthma exacerbations, but contributed to increased FEV1 in subgroups with less than 70% of FEV1 baseline. In addition, enrolled RCTs showed significant baseline heterogeneity in both vitamin D dose and demographic characteristics.

There are still no objective markers to assess asthma severity. Although asthma patients have a certain degree of the inflammatory response, some severe patients may also develop exacerbation and deterioration of asthma after inflammation is controlled (84). The Primary Care Asthma Control Screening tool (adult) (85) or the Asthma Control Test [adults (86) or children (87)] can be used to quickly assess control of asthma symptoms with questionnaires. Clinical efficacy results indicated a cutoff point of 19 or lower for C-ACT or ACT (86, 87), indicating incomplete asthma control. Over the years, the definition of acute asthma or exacerbation has varied. Currently, severe exacerbation is defined as requiring corticosteroid use for at least 3 days or as an inpatient or emergency room visit due to the need for corticosteroids for asthma. Moderate deterioration was defined as an event that required modification of treatment to prevent it from becoming severe and not so severe as to require oral corticosteroids (OCS) by the American Thoracic Society/European Respiratory Society (88). The transcriptomic profile of bronchoscopy has identified high and low type 2 immunity asthma and other molecular phenotypes (89, 90). Adaptive T helper 2 cell activation produces a series of cytokines following allergen sensitization and stimulation of dendritic cells. Eosinophils are recruited to the lung mucosa by chemokine receptors and other eosinophil chemo-attractants (3). In non-eosinophilic asthma, innate lymphoid cells, macrophages, and neutrophils have an important role in stimulating the release of cytokines (interleukin-33 and interleukin-25) or chemokines (C–X–C motif chemokine ligand 8), to regulate the immune response (91, 92).

With the development of economy and medical level, vitamin D, a proline obtained from skin exposure to ultraviolet B (UVB) light and dietary intake from the liver, fish, egg yolk, and other sources, is transformed to 25-hydroxyvitamin D [25(OH)D] in liver (93, 94), which has gradually attracted the attention of the majority of domestic and foreign research scholars. Several studies have demonstrated a correlation between vitamin D deficiency and asthma prevalence and severity. Patients with vitamin D deficiency have a higher prevalence of asthma, which could be a strong prediction factor of asthma (95–97). Additionally, vitamin D deficiency was also associated with severe asthma exacerbations in multiple prospective and retrospective (98–100). Compared with children with insufficient or sufficient vitamin D, there was a correlation between vitamin D deficiency and pulmonary dysfunction in asthmatic children treated with inhaled corticosteroids (101). Although as a nutrient that regulates metabolism, vitamin D has been shown to immunomodulate various immune cells and structural cells in the airway, by activating vitamin D receptors (VDR) (102–105). Several *in vitro* and *in vivo* studies using asthma murine models have also shown that vitamin D modulated the inflammatory response. In vitamin D-treated asthmatic mice, the Penh values, type 2 cytokines, perivascular and peribronchial inflammation, goblet cell proliferation, total IgE and histamine, and mucus hypersecretion were all significantly reduced (106). Vitamin D deficiency also potentiated oxidative stress and corticosteroid resistance in severe asthma exacerbations. Vitamin D3 supplementation significantly increased the change of FEV1, and effectively alleviated ROS and DNA damage, which were related to a decrease in TNF-α and NF-κB in epithelial cells (107). Oxidative stress-activated transcription factors (TF) and signaling pathways, and partly activated the innate immune response through toll-like receptors 2 (TLR-2) and toll-like receptors 4 (TLR-4), thus promoting the release of cytokines and chemokines. In addition, oxidative stress had an important role in affecting corticosteroid insensitivity by inhibiting the activity and expression of HDAC-2 via serine hyperphosphorylation (108). Although there has been sufficient evidence that vitamin D deficiency was associated with progression and exacerbation of asthma, there are many inconsistencies in multiple prospective clinical studies. The researches indicated that vitamin D supplementation was not of use in preventing severe asthma exacerbations or control of asthma in children (73, 82, 109) or adults (76, 78). High-dose vitamin D supplementation during pregnancy did not reduce the risk and improve the allergy outcomes of asthma in children (under 6 years of age) compared to standard doses (110).

Additionally, we further confirmed that it could effectively alleviate the probability of asthma exacerbations in children and when follow-up time was less than 6 months (Figure 6). Noticeably, it significantly enhanced FEV1 in patients whose FEV1 baseline value was less than 70%, though there was only one study included in the subgroup. Only one former meta-analysis (111) demonstrated that vitamin D supplementation couldn’t reduce asthma exacerbations and FeNO, nor could it improve lung function and asthma symptoms. Our meta-analysis offers several advantages over previous meta-analyses. First, all included studies were RCTs, and the studies with incomplete data were strictly excluded according to the standard. Second, subgroup analyses of included studies were performed to minimize heterogeneity for baseline values including age, FEV1 values, and follow-up time in our analysis (Table 2). Finally, the sensitivity analysis was similar to the above results, indicating that the results of this meta-analysis were reliable (Figure 7). However, there are still many defects in our meta-analysis. First, heterogeneity in dose and mode of administration of vitamin D in enrolled studies was unavoidable, and not all subjects enrolled in various studies received consistent basic anti-asthma therapy. Some studies standardized the therapeutic dose of glucocorticoids for asthma (74, 76, 77, 80, 81), some observed it as an outcome variable (73, 78, 82, 83). And most studies didn’t mention whether the hormone dose was changed during the follow up (73, 78, 79, 82), so we are not sure whether this will affect the accuracy of the results of RCTs. Second, the sample size of several studies included in this analysis was too small to demonstrate the reliability of the results. Finally, not all subjects enrolled in the study were asthmatics of the same severity or etiology.

In conclusion, our meta-analysis demonstrated that there was high heterogeneity in RCTs regarding improvement in exacerbation of asthma and FEV1 with vitamin D supplementation. Vitamin D supplementation led to a reduction of asthma exacerbations, especially in children and with a follow-up period of less than 6 months. In addition, it played an important role in improving FEV1 in patients with FEV1 baseline values below 70%. Though evaluating the ACT scores and FENO, we found that vitamin D worked the same way as a placebo. Based on the results of the GRADE analysis, all major findings were low or very low except for the FEV1 subgroup with baseline values below 70%. Therefore, a larger and well-designed RCT is needed to evaluate the effect of vitamin D in the treatment of asthma, including uniform vitamin D dosing and administration mode, follow-up time, and strict inclusion and exclusion criteria. Furthermore, whether basic asthma treatment should be standardized during follow-up or used as an outcome measure of asthma treatment efficacy still needs to be further explored.

## Data Availability Statement

The original contributions presented in this study are included in the article/supplementary material, further inquiries can be directed to the corresponding author.

## Author Contributions

ML: data selection, data extraction, quality assessm statistical analysis, and writing – original draft. JW: data selection, data extraction, and quality assessment. XS: conceptualization, writing – review, and supervision.

## Conflict of Interest

The authors declare that the research was conducted in the absence of any commercial or financial relationships that could be construed as a potential conflict of interest.

## Publisher’s Note

All claims expressed in this article are solely those of the authors and do not necessarily represent those of their affiliated organizations, or those of the publisher, the editors and the reviewers. Any product that may be evaluated in this article, or claim that may be made by its manufacturer, is not guaranteed or endorsed by the publisher.
